# A PiggyBac mediated approach for lactoferricin gene transfer in bovine mammary epithelial stem cells for management of bovine mastitis

**DOI:** 10.18632/oncotarget.22210

**Published:** 2017-10-31

**Authors:** Neelesh Sharma, Do Luong Huynh, Sung Woo Kim, Mrinmoy Ghosh, Simrinder Singh Sodhi, Amit Kumar Singh, Nam Eun Kim, Sung Jin Lee, Kafil Hussain, Sung Jong Oh, Dong Kee Jeong

**Affiliations:** ^1^ Department of Animal Biotechnology, Faculty of Biotechnology, Jeju National University, Jeju, Republic of Korea; ^2^ Division of Veterinary Medicine, Faculty of Veterinary Science & Animal Husbandry, Sher-e-Kashmir University of Agricultural Sciences & Technology of Jammu, Jammu, India; ^3^ Animal Genetic Resources Station, National Institute of Animal Science, Rural Development Administration, Namwon, Republic of Korea; ^4^ Department of Animal Biotechnology, College of Animal Bioscience and Technology, Kangwon National University, Chuncheon, Republic of Korea; ^5^ National Institute of Animal Science, Wanju-gun, Republic of Korea

**Keywords:** antibacterial milk peptide, bovine lactoferricin, PiggyBac, bovine mastitis, antibacterial activity

## Abstract

The antibacterial and anti-inflammatory properties of lactoferricin have been ascribed to its ability to sequester essential iron. The objective of the study was to clone bovine lactoferricin (*LFcinB*) gene into PiggyBac Transposon vector, expression study in the bovine mammary epithelial stem cells (bMESCs) and also to determine the antimicrobial property of recombinant *LFcinB* against bovine mastitis-causing organisms. The PiggyBac-LFcinB was transfected into bMESCs by electroporation and a three fold of LFcinB secretion was observed in the transfected bMESCs medium by ELISA assay. Furthermore, the assessment of antimicrobial activity against mastitis causing pathogens *Staphylococcus aureus* and *Escherichia coli* demonstrated convincing evidence to prove strong antibacterial activity of LFcinB with 14.0±1.0 mm and 18.0±1.5 mm zone of inhibition against both organisms, respectively. The present study provides the convincing evidence to suggest the potential of PiggyBac transposon system to transfer antibacterial peptide into bMESCs or cow mammary gland and also pave the way to use bovine mammary gland as the bioreactors. Simultaneously, it also suggest toward commercial utilization of LFcinB bioreactor system in pharmaceutical industry.

## INTRODUCTION

Infectious diseases constitute a pertinent concern in Human and Veterinary Medicine worldwide. Bovine mastitis is an inflammatory disease of udder caused by multietiolgical agents but *Staphylococcus aureus* (*S. aureus*) and *Escherichia coli* (*E. coli*) are primarily responsible for the immensity of bovine mastitis cases [1, 2]. *S. aureus* is a normal inhabitant of the udder [3], and *E. coli* is found in the animal’s environment. The unbounded use of antimicrobials to hamper microbial growth have bestowed to the development of bacterial resistance against a broad spectrum of antibiotics.

Lactoferrin (LF) is a multifunctional protein found in bovine milk [4] with an antimicrobial properties: it is active against many Gram-negative and Gram-positive bacteria [5], viruses [6], and various types of fungi and parasites [7, 8]. Owing to its antibacterial activity, that would be a promising candidate to replace conventional therapy used for bovine mastitis treatment. Various therapeutic properties of LF have been described in details in a recent review paper [9]. The antimicrobial peptides (AMPs) in modern pharmaceutical research play the vital role for human and veterinary medicine. These peptides show a broad spectrum of antimicrobial activity and can also trigger specific defense responses in the host. The earlier evidence suggested that AMPs showed high effectiveness against pathogens in mastitic bovine milk and low efficiency in milk from healthy cows. In present study, the functional potential of antibacterial peptide LFcinB in inflammatory responses and the expression profiling in bovine mammary epithelial stem cells was investigated. Moreover, the AMPs present the advantage of being derived from a harmless and inexpensive source and have therefore an undeniable potential for use in medicine [10].

Numerous theories have been postulated on the antibacterial mechanism of action of AMPs and found that they exert their action on the cytoplasmic membrane of susceptible microorganisms [11]. Later different molecular mechanisms of membrane perturbation by antimicrobial peptides have increased our understanding toward the most exact mechanism of action [11, 12] viz. pore formation on the bacterial membrane by thinning the membrane or destabilizing the membrane bilayer [13, 14]. Other theories suggested that AMPs have bactericidal activity through inhibition of macromolecule biosynthesis or by interacting with specific vital components inside the microorganism, or both [15].

Bovine lactoferricin (LFcinB) is a peptide fragment produced by acid-pepsin hydrolysis of lactoferrin obtained from cow’s milk [16]. LFcinB, which consists of 25 amino acid residues (17 to 41) proximal to the N-terminus of bovine lactoferrin, is notable for its ability to bind iron and relatively have high proportion and asymmetrical clustering of basic amino acid residues. LFcinB has attracted considerable interest because of its well established antimicrobial activity [17, 18]. Owing to its potential antimicrobial and immunomodulator activity, it is necessary to produce more this bioactive peptide from the mammalian system for further studies and clinical trials. Therefore, this study was aimed to increase antibacterial ability of mammary epithelial cells through increased secretion of the antibacterial agent (LFcinB) and to provide strong innate udder immunity to fight against intramammary infections. The present study was focused on the synthesis of a fragment from 17 - 41 amino acids and to investigate the ability of bovine mammary epithelial stem cells for expression, secretion, production and antibacterial activity of LFcinB to combat with major mastitis causing organisms. Due to high milk production ability, bovine mammary gland can be used as bioreactors, and are supposed to be the feasible tools for the production of LFcinB in large scale for pharmaceutical industry. The construction of an efficient and specific eukaryotic expression vector is a key regulatory point for the development of a mammary gland as a bioreactor.

To the best of our knowledge, this is the first report on the heterologous expression of the hybrid antibacterial peptide LFcinB into PiggyBac system with high antibacterial properties against bovine mastitis origin *S. aureus* and *E. coli* and also provides the basis for next cost-effective expression of other antimicrobial peptides in genetic engineering. Recent genetic studies have also suggested that PiggyBac can be used as potential alternate of viral vectors for the gene therapy and transgenic production.

## RESULTS

### Construction of PiggyBac-LFcinB recombinant

**Figure 1:**
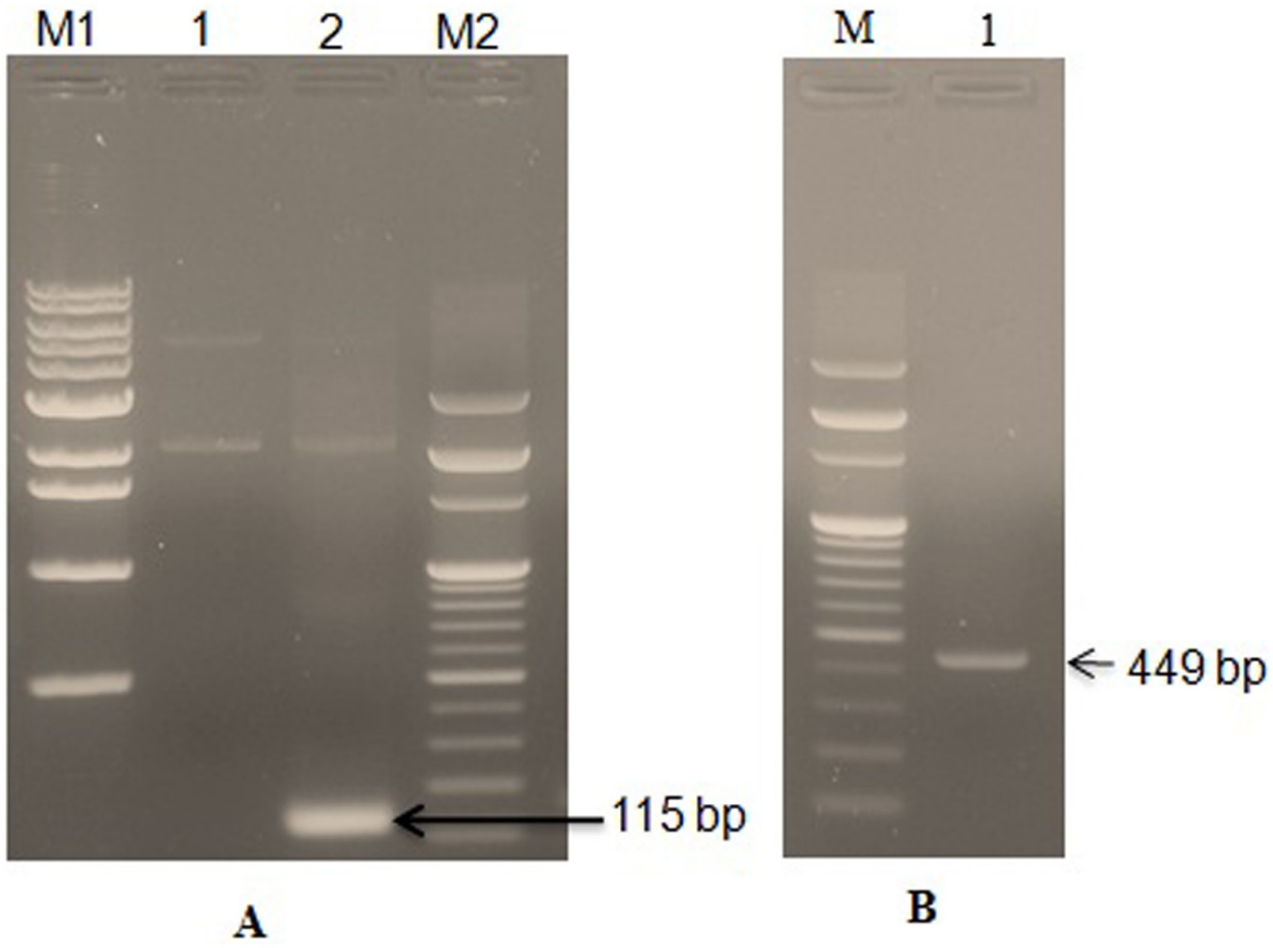
PCR detection and amplification of LFcinB gene in the pGEM-B1 plasmid **Panel A**: PCR detection of bovine lactoferricin gene in the pGEM-B1 vector with detection primer. Lane M1- 1 kb DNA ladder; Lane 1- pGEM-B1 vector with *LFcinB* gene; Lane 2- PCR detected *LFcinB* gene (115 bp) and Lane M2- 100 bp DNA ladder. **Pane**l **B**: PCR amplification of bovine lactoferrin gene from the pGEM-B1 vector using cloning primer. Lane M- 100 bp DNA ladder and Lane 2- PCR amplified *lactoferrin* gene (449 bp).

**Figure 2:**
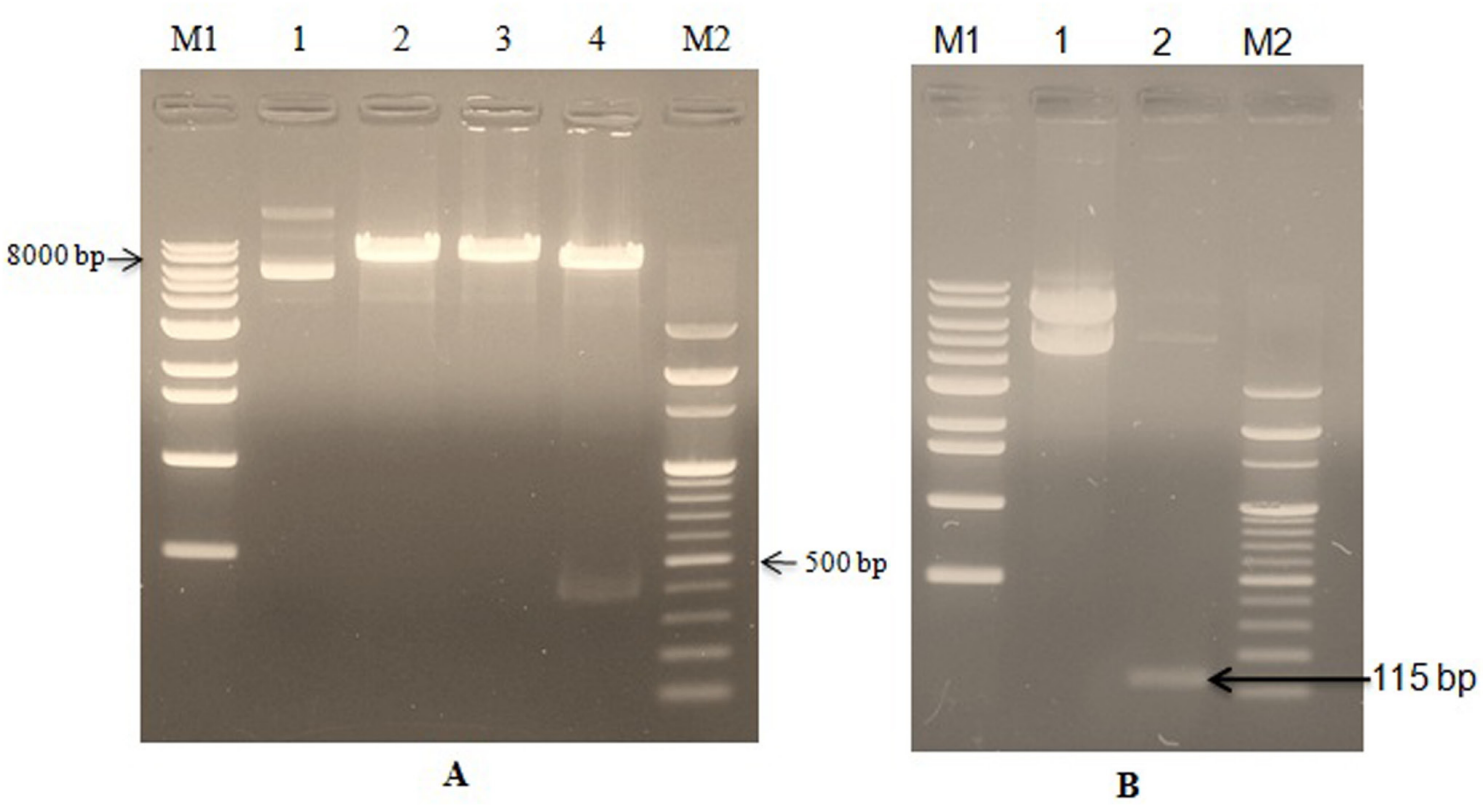
Confirmation of bovine lactoferricin ligation in the recombinant PiggyBac-LFcinB plasmid by restriction enzymes and PCR **Panel A**: Confirmation of bovine lactoferricin ligation by restriction enzymes. Lane M1- 1Kb DNA marker; Lane 1- Undigested recombinant plasmid (PiggyBac-LFcinB); Lane 2- Single digestion of recombinant plasmid with *BamHI* ; Lane 3- Single digestion of recombinant plasmid with *EcoRI* ; Lane 4- Double digestion of recombinant plasmid with *BamHI* and *EcoRI* ; Lane M2- 100 bp DNA ladder. **Panel B**: Confirmation of bovine lactoferricin ligation by PCR. Lane M1- 1 kb DNA marker; Lane 1- Undigested recombinant plasmid (PiggyBac-LFcinB); Lane 2- PCR detected LFcinB gene (115 bp); Lane M2- 100 bp DNA marker.

The pGEM-B1 plasmid containing LFcinB was identified using bovine lactoferricin detection primer (Figure 1A). The amplified gene fragment of 449 bp was visualized on 1.5% agrose gel electrophoresis (Figure 1B). The expression vector PiggyBac was digested with the restriction enzymes *EcoRI* and *BamHI*. The amplified fragment of the synthetic gene was ligated into PiggyBac vector and the recombinant fragment was named as PiggyBac-LFcinB. The gene products were popped out by the restriction digestion to confirm the presence of the gene fragments in the respective vector (Figure 2A). Simultaneously, the incorporation of the gene into 106 the vectors was confirmed by PCR amplification from the recombinant plasmid with specific forward and reverse primers (Figure 2B). The length of 449 bp suggested ligation of gene of interest into the PiggyBac vector.

The quality of the recombinant was confirmed by DNA sequencing and clone was evaluated using sequencing chromatogram. The result has been shown by using Finch TV software, and the sequence was evaluated again by BLASTn (www.blast.ncbi.nlm.nih.gov/Blast.cgi?PAGE_TYPE=BlastSearch). It was found that the sequence data has shown 100% similarity with original gene sequence with the 449 bp length. Therefore, there is no doubt that the quality of the clone produced is under best quality score.

### LFcinB transfected stable cell lines

**Figure 3:**
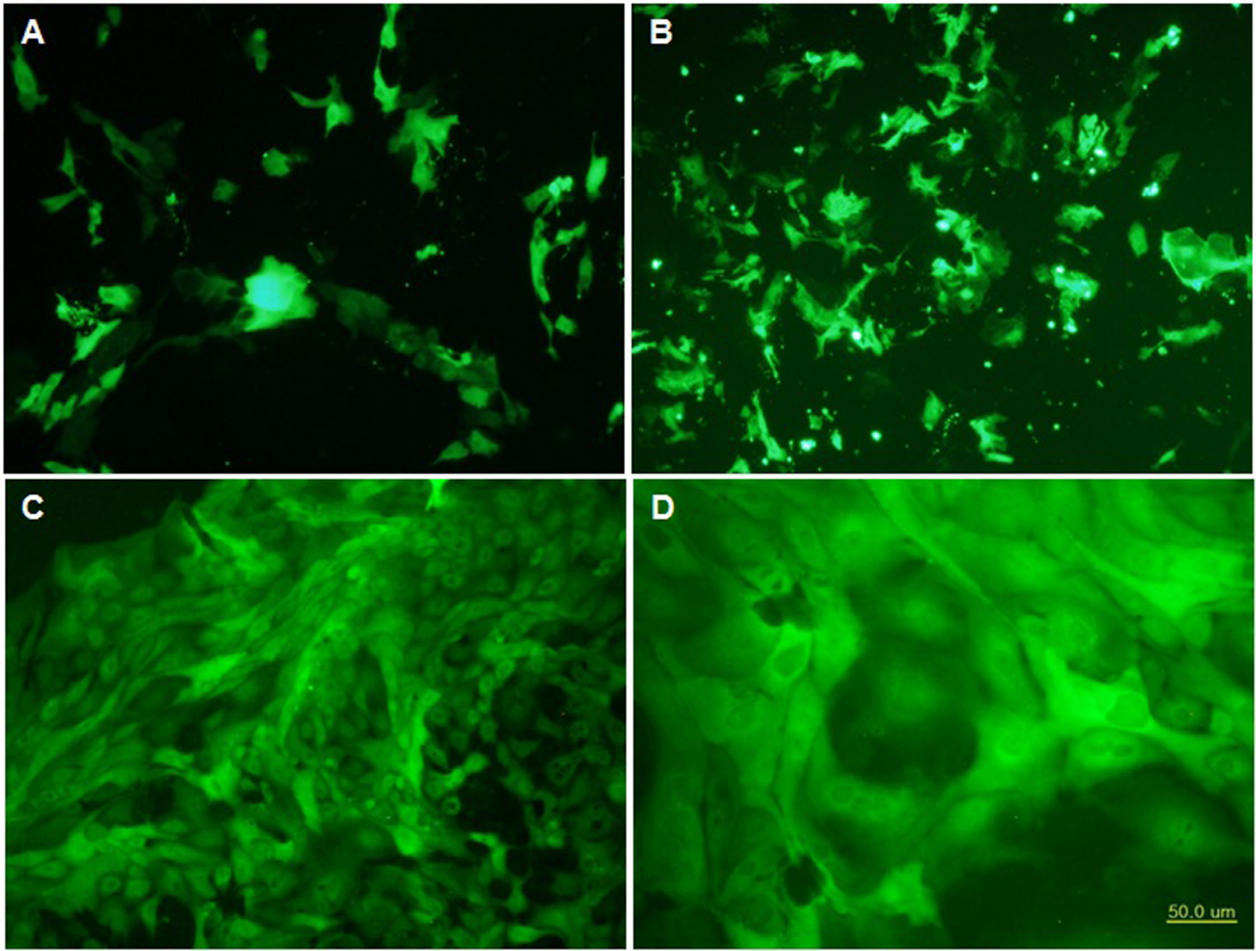
Transfection, expression and establishment of bovine mammary epithelial stable cell lines with recombinant PiggyBac-LFcinB **(A)** Expression of Pmax GFP (positive control) after 24 h of transfection (100x); **(B)** Expression of recombinant bovine lactoferricin GFP after 24 h of transfection (100x); **(C)** Purified recombinant bovine lactoferricin cell line after 14 days of transfection in puromycin as selection marker (100x); **(D)** Purified recombinant bovine lactoferricin cell line after 14 days of transfection in puromycin which was used as selection marker at high resolution (200x). Cells showed the clear morphology of bovine mammary epithelial stem cells with nucleus and nucleolus.

The transfection results demonstrated the expression of Green Fluorescent Protein (GFP) of recombinant bovine lactoferricin after 48 h of transfection with 15-20% transfection efficiency in the bMESCs (Figure 3A), whereas positive control Pmax GFP showed about 30-35% (Figure 3B). After 48 h transfection, cells were subjected for selection with 250 μg/ml puromycin as a selection marker in mammalian cells and purified colonies were obtained within 2 weeks (Figure 3C, 3D). Puromycin resistant pure cells were picked up and transferred to fresh medium for multiplication and finally LFcinB purified cells were obtained. All these in together suggest that the utilization of PiggyBac system is exceeding compared to normal vectors. That partly demonstrated that PiggyBac is one of a powerful tool for transfection of mammalian cells. The purified transfected cells were used for further analysis and determination of lactoferricin production.

### Transfectants produce high quantity of LFcinB in the medium

**Figure 4:**
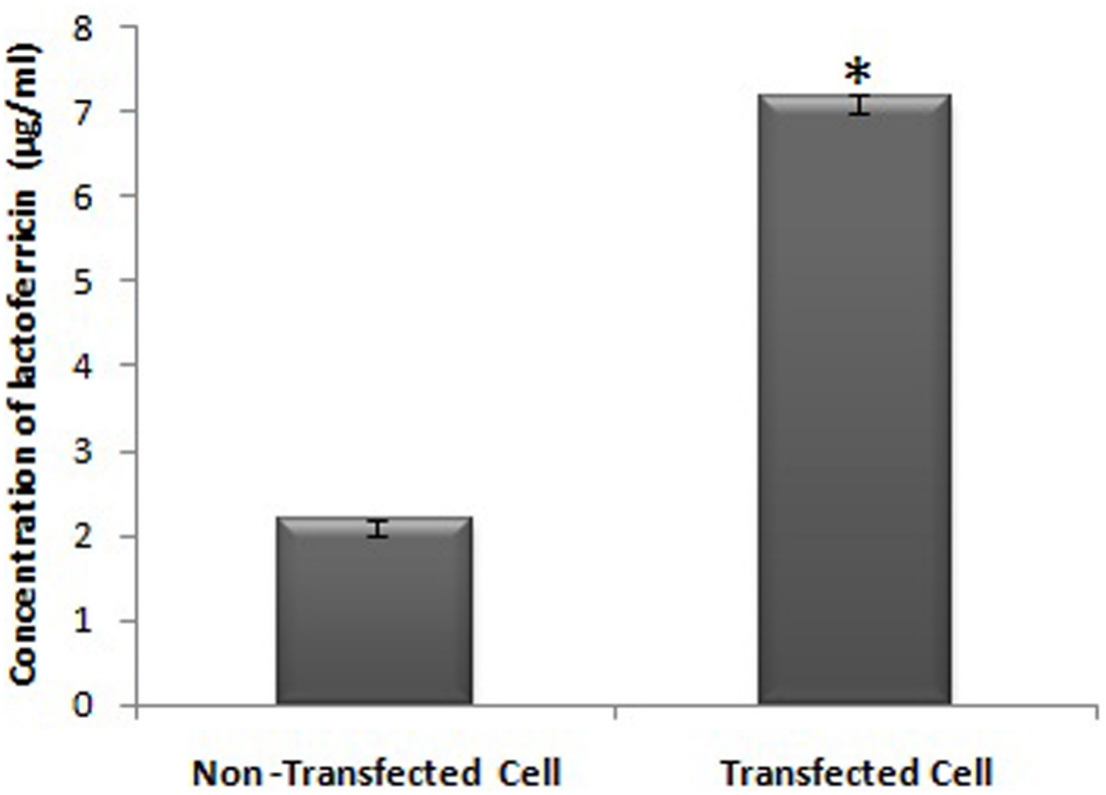
Bovine lactoferricin secretion ability of transfected cells and detection of its concentration in the cell culture medium of transfected and non-transfected bMESCs (P ≤ 0. 05)

The dramatic increase of LFcinB secretionwas noticed in the transfected BMESCs in *in-vitro* culture system. In the non-transfected cell, the concentration of LFcinB in the medium was found to be 2 μg/ml whereas transfected cells had three folds higher LFcinB production ability (Figure 4). It suggests that the LFcinB transfected cells may be used as good source of LFcinB with antibacterial property and can protect mammary gland from intramammary infections by providing innate immunity. The increased production of LFcinB has gained a significant interest to study the antibacterial efficacy of LFcinB in transfected cells media as such.

### Detection of *LFcinB* in the stable cell lines by immunocytochemistry

**Figure 5:**
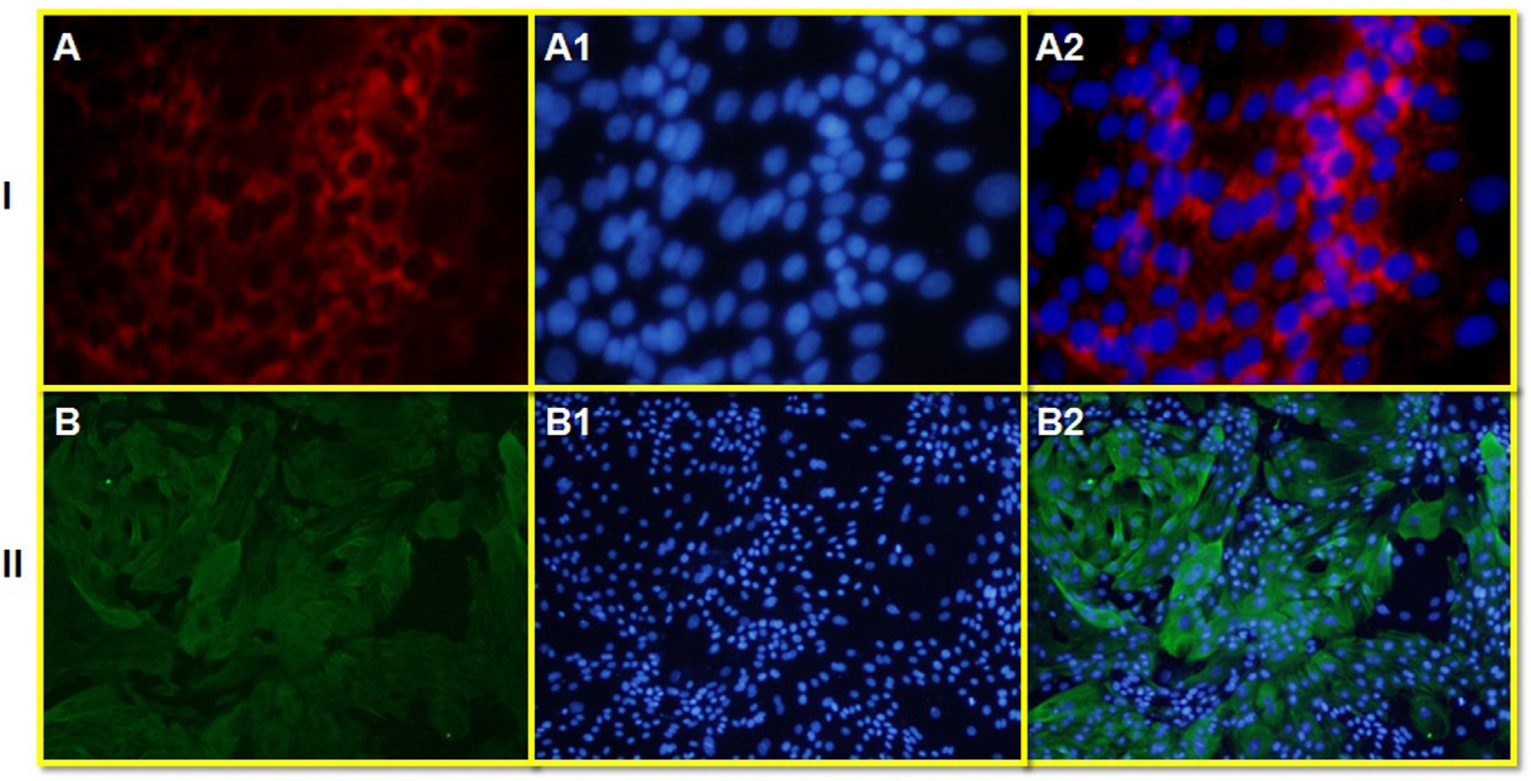
The presence of red fluorescence signals of bovine lactoferricin primary antibody as a marker of successful transfection in cultured bovine mammary epithelial stem cells (bMESCs) **Panel I**: Transfected bMESCs immunostained with mouse primary antibody anti-Bovine Lactoferricin and goat anti-mouse secondary antibody conjugated with Phycoerythrin (PE) showing red fluorescence **(A)**, DAPI nuclear staining **(A1)** and merged **(A2)** (100x). **Panel II**: Transfected bMESCs immunostained without primary antibody (as control) showing only green signals of recombinant LFcinB, while no signals of goat anti-mouse secondary antibody conjugated with Phycoerythrin (PE) **(B)**, DAPI nuclear staining **(B1)** and merged **(B2)** (200x).

The purified colonies of LFcinB transfected cells were subjected to immunochemical analysis for further confirmation of cloning and transfection. Transfected cells were immunostained with anti-bovine lactoferricin antibody and further staining with phycoerythrin conjugated anti-mouse antibody emitted red fluorescence under microscope (Figure 5I) and confirmed the successful transfection of LFcinB and its expression into bMESCs. Figure 5II shows green fluorescence signals of the recombinant LFcinB clone (GFP) as a control without staining with primary antibodies and thus confirmed the transfection. These results further confirmed the successful cloning of LFcinB and the potential of PiggyBac to express the insert in transfected bMESCs.

### Antibacterial activity of *LFcinB*

**Figure 6:**
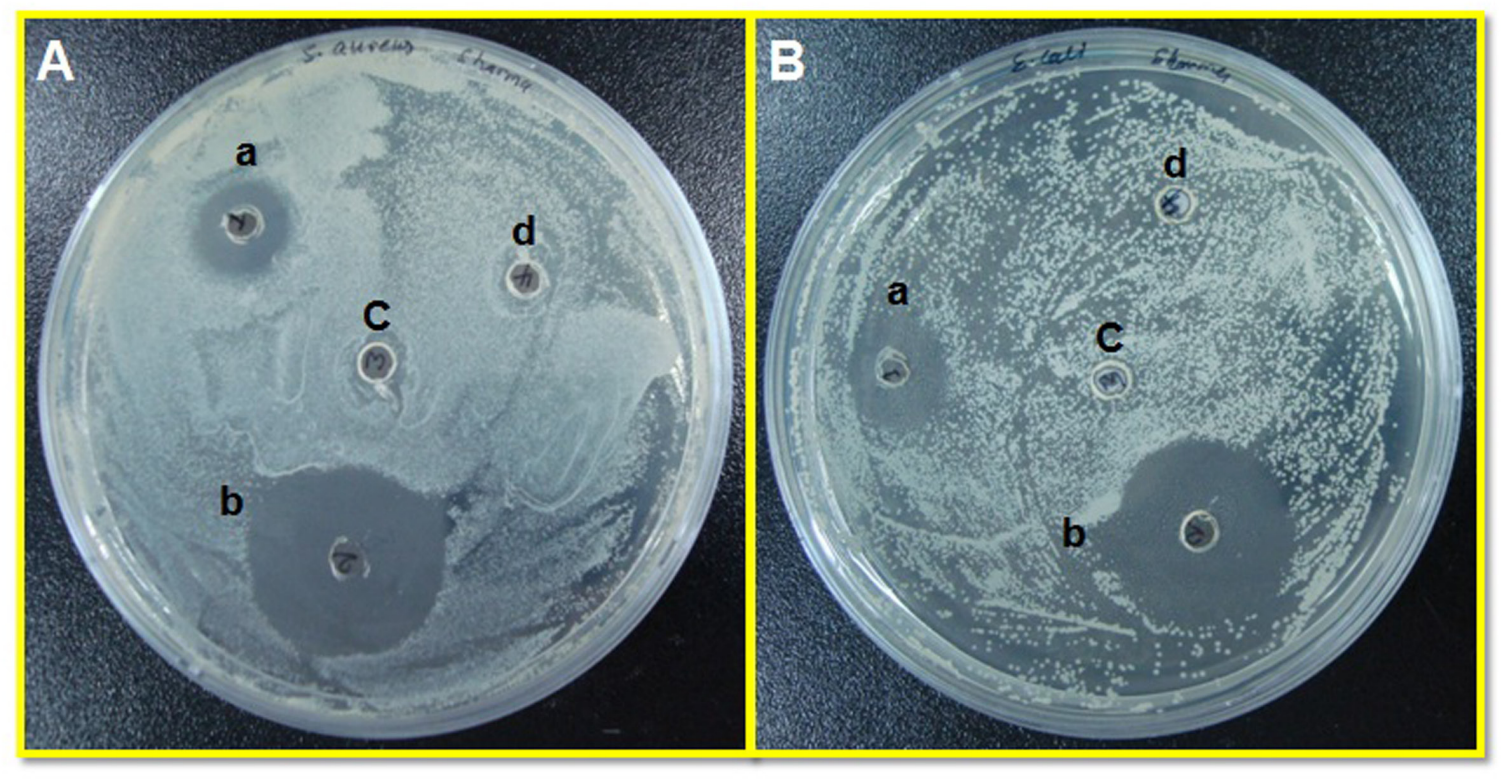
Evaluation of antibacterial activity of recombinant LFcinB against bovine mastitis-causing Gram positive and Gram negative bacteria by agar well diffusion assay Plates showing susceptibility to LFcinB secreted by transfected cells in media (a) against both *S. aureus* **(A)** and *E. coli* **(B)**. A strong zone of inhibition with Penicillin-Streptomycin (50 μg/ml) as positive control (b) and no zone of inhibition with media from non-transfected cells (c) and PBS (d) as negative controls.

Antibacterial activity for produced LFcinB was assessed by agar well diffusion assay as are shown in Figure 6. LFcinB exhibited higher antibacterial efficacy against *E. coli* compared to *S. aureus*. The zone of inhibition observed as a result of LFcinB activity against *S. aureus* growth was 14.0 ± 1.0 mm (Figure 6Aa), while that for standard antibiotic (positive control) was 23.7 ± 1.5 mm (Figure 6Ab). There was no growth inhibition in negative controls i.e. phosphate buffer saline (PBS; Figure 6Ac) and media from non-transfected cells (Figure 6Ad). Similar trend were observed for *E. coli*, where LFcinB had 18.0 ± 1.5 mm (Figure 6Ba) zone of inhibition and were comparable to antibiotic positive control (23.1 ± 0.9 mm) (Figure 6Bb) while no inhibition was observed in control wells (Figure 6Bc, 6Bd). The results of micro-dilution assay and agar-well assay were strong indicative of the antibacterial activity of bovine lactoferricin.

**Figure 7:**
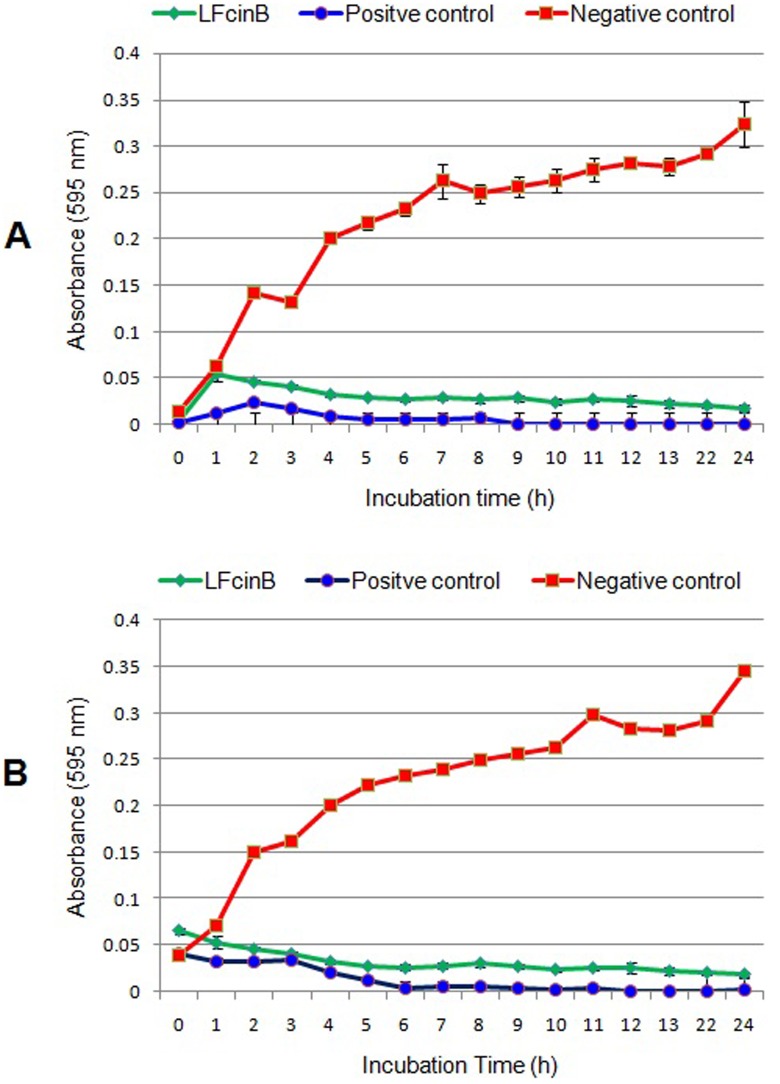
Antibacterial kinetic study of bovine lactoferricin against mastitis causing bacteria by broth micro well assay *S. aureus* **(panel A)** and *E. coli* **(panel B)** (1 x 10^5^ CFU) were treated with media from recombinant PiggyBac-LFcinB transfected and non-transfected cells, incubated at 37°C for 24 h. Penicillin-streptomycin (50 μg/ml) was used as positive control. Optical density of bacterial growth at different time intervals.

The antimicrobial kinetic study of recombinant bovine lactoferricin was performed by broth microwell assay, and the growth of bacteria was determined at different time points. Data obtained from these assays established that LFcinB has strong antibacterial activity against both *S. aureus* (Figure 7A) and *E. coli* (Figure 7B) when compared to control. Absorbance values obtained from LFcinB treated group were compared with those obtained for the standard antibiotic treated group against both isolates. The results of the kinetic study clearly confirmed the antibacterial activity from 4 to 5 h onward.

## DISCUSSION

Bovine lactoferricin is a 25 amino acid residue peptide released from lactoferrin upon pepsin digestion [19]. Lactoferrin is the major iron binding protein in milk of many mammalian species, has shown strong cationic antimicrobial property against a wide range of microorganisms including Gram-positive and Gram negative [20-24]. Earlier studies have confirmed that lactoferrin may have therapeutic potential in mastitis [25, 26]. Moreover, Wall et al. [27], has been produced a genetically enhanced cows resist intramammary *Staphylococcus aureus* infection with targeting lysostaphin transgene. Transgenesis has been suggested as a means to combat mastitis [28]. Recent studies have been used lactoferricin as anti-bacterial agent against diarrheal organisms (*S. aureus* and *E. coli*) [29, 30].

Lactoferricin is widely distributed, but it is difficult and time-consuming to isolate lactoferricin directly from natural sources. Therefore, it would be an imperative and worth trying to realize the expression and purification of recombinant lactoferricin with low cost and high bioactivity via modern biotechnology. Zhang et al. [18], used plasmid-mediated gene transfer technique to enable mammary cells to synthesize and secrete bovine lactoferricin and bovine tracheal antimicrobial peptides to prevent bovine mastitis.

The present study was focused on the antibacterial activity of lactoferricins in bovine mammary epithelial stem cells against major mastitis causing bacteria. The LFcinB gene was cloned into the PiggyBac vector system. We transfected the PiggyBac- LFcinB clone *in vitro* into bMESCs and results were analyzed by immunocytochemistry. The strong GFP expression and immunochemistry results have shown that the gene has been successfully integrated into the genome of transgenic cells and highly expressed.

Transposable elements have been routinely used for genetic manipulation in lower organisms. In contrast, the usage of transposons in vertebrate systems is still limited [31]. Because of low transfection or hard to transfect primary mammary epithelial cells, researchers are using viral transfection systems. Hence it was decided to move one step further by using non-viral vector with potential transfection efficiency in bMESCs. Long-term expression of a transgene is most reliably achieved by the stable integration. Retroviral and lentiviral vectors have been used for this purpose, but their cargo capacity is limited to 10 kb, and they are not suited for the delivery of intron-containing cargos. Moreover, most important is that these viral systems have an immunogenic and tumorigenic potential [32]. The PiggyBac transposase is an efficient gene transfer vector active in a variety of cell types and proven to be amenable to modification [33]. The recent study has shown the efficient application of PiggyBac mediated transposition vector in the germ cells of chicken [34] and production of bovine induced pluripotent stem cells (iPSCs) [35]. The DNA transposon PiggyBac is widely used as an efficient tool in mammalian experimental systems for transgenesis, mutagenesis and genome engineering [36]. This study showed that PiggyBac is more efficiently expressed in mammary cell lines of bovine and is amenable to further molecular modification without reducing its activity [37]. The present research work provides a powerful approach to enhance the properties of the PiggyBac system for further applications such as genetic engineering and gene therapy in livestock.

Designing and insertion of a new hybrid peptide PiggyBac-LFcinB in between BamHI and EcoRI lead to formation of recombinant expression vector for heterologous expression of PiggyBac-LFcinB in *E. coli* top10. The recombinant showed antimicrobial activity assayed by micro-dilution and agar-well diffusion methods. Bi *et al.* [38], demonstrated the bovine lactoferricin antimicrobial activity assayed by agar diffusion test only. Lactoferricin was shown to interact with lipopolysaccharide (LPS) of the Gram-negative bacterial membrane of *E. coli* [39] hence it has high antibacterial activity against *E. coli* [40]. Our findings also showed the comparatively higher antibacterial property of bovine lactoferricin against *E. coli* in comparison to *S. aureus*. Kutila et al. [41], have reported antibacterial property of bovine lactoferrin against bovine udder pathogens. Simojokia et al. [42], have been reported that high concentration of human lactoferrin in milk of rhLf-transgenic cows relieves signs of bovine experimental *Staphylococcus chromogenes* intramammary infection. While a study on recombinant human lactoferrin (rhLf) transgenic cows in an *E. coli* mastitis model and reported inconclusive results [43]. They have reported that rhLf-transgenic cows showed milder systemic signs and lower serum cortisol and haptoglobin concentrations than did controls. However, Lf does not seem to be a very efficient protein for genetic engineering to enhance the mastitis resistance of dairy cows. But mastitis is caused by not only due to *E. coli*, hence it could therapeutic effect in other type of mastitis as the report of other researcher [42].

The most striking observation have been reported by Lacasse et al. [44], that Lf increases the inhibitory activity of penicillin up to 4-fold in most penicillin-susceptible *Staphylococcus aureus* strains, whereas this increase was 4- to 16-fold in penicillin-resistant strains.

Nuclear magnetic resonance (NMR) study of Hwang et al. [45], demonstrated that free LFcinB adopts a conformation in an aqueous solution that is quite different from that found in the intact protein. During this phase, peptide loses the α-helix seen in intact bovine lactoferrin (bLF) and forms a twisted β-sheet and resulting peptide becomes markedly amphipathic. The property of LFcinB to form amphipathic structures with clear hydrophobic and positively charged faces is a characteristic antibacterial trait they share with other peptides that display antimicrobial activity. Now it is well established that iron-independent mechanisms such as direct interaction with bacteria leading to membrane destabilization, modulation of bacteria motility, aggregation or endocytosis into host cells, inhibition of adherence and biofilm formation are responsible for the antimicrobial property of lactoferricin [46].

Lactoferricin (LFcin) antimicrobial potential is conferred in its ability to perturb membrane structure and it also act in synergism with other microbial agents. LFcin exhibits numerous biological activities in common with those of lactoferrin (LF). Whereas LFcin suppresses the activation of innate immunity by microbial components such as lipopolysaccharide (LPS) and CpG DNA, the peptide itself activates immunity. Administration of LFcin analogs has been shown to protect the host via direct antimicrobial activity and immunostimulatory effects in several infection models [47]. Ellison *et al.* [48], have shown other synergistic effects: the secretory IgA–Lf complex from human milk has bactericidal activity against both Gram-positive and Gram-negative bacteria. Thus oral immunization by mothers may help protect their infants.

For many years, the antimicrobial activity of lactoferricin was attributed to their ability to sequester iron thereby depriving potential pathogens of this essential nutrient. Our findings raise the possibilities to develop the cows with an effective antimicrobial defense system in the udder compartment after transplanting LFcinB transfected bMESCs to provide strong udder immunity against mastitis causing organisms. Owing to antibacterial activity of LFcinB that would be a promising candidate to replace conventional therapy of mastitis in dairy cows using antibiotic, which has been identified to be related to severe antibiotics resistance. Present findings may also open the possible use of bovine mammary gland as the non-genetically modified organism (non-GMO) bioreactor for the bulk production of proteins of pharmaceutical interest. A Recent study has been tried to use bovine mammary gland as a bioreactor for the production of human lactoferrin, but they generated transgenic cattle [49].

Of particular significance in the context of this report is the ability to introduce transgenes into bovine mammary stem cells to produce proteins of interest, including those usually present in cow milk, is an important discovery. This finding could have many applications in biotechnology considering the high milk output that can be obtained from high milk producing breeds of cattle and the ease of protein recovery from secreted milk.

LFcinB advantage of being a broad antibacterial spectrum without inducing resistance against antibiotics, amplifies its potential for use as an alternative to widely used antibiotics in the management of bovine mastitis, and would be the cost effective for the production of LFcinB in large scale for pharmaceutical industry. Large-scale production of biopharmaceuticals by current bioreactor techniques is limited by low transgenic efficiency and low expression of foreign proteins [49]. It could partly replace the use of antimicrobials, which cause problems due to their presence in the form of residues in milk and therefore, potentiate the risk for emergence of resistance.

## MATERIALS AND METHODS

### Reagents and chemicals

All the chemicals used in the preparation of reagents, buffers, and the preparation of bacterial culture media (broth and agar) were of analytical grade purchased from Becton, Dickison and Company, France except sodium chloride from Georgiachem Co. Korea and Brain Heart Infusion (BHI) broth from Acumedia, Neogene, Scotland, UK. The restriction enzymes and T4 DNA ligation kit were purchased from Enzynomics, Korea and Invitrogen, USA, respectively. Primers were synthesized by Cosmo Genentech, Korea. The Exprep plasmid SV mini plasmid purification kit and Expin Gel DNA gel extraction kit were procured from Gene All Biotechnology, Korea. PCR cleanup kit was purchased from Genet Bio, Korea. Bovine mammary epithelial stem cells were characterized and preserved in our lab. All plastic-wares including cell culture flasks and plates were purchased from Nunc, Thermo Scientific, Korea. Dulbecco’s Modified Eagle’s Medium/F12 (DMEM/F12), penicillin-streptomycin and growth factors were purchased from Gibco, USA and Sigma, USA respectively. Fetal calf serum (FBS) was purchased from Hyclone, USA. Total easy-Blue RNA extraction kit and *E. coli* Top10 competent cells were procured from Intron Biotech, Korea. Puromycin and Ampicillin were purchased from Georgiachem Co. Korea.

### Bacterial strains, plasmids and growth conditions

Plasmid cloning vector PiggyBac was procured from Clontech, USA (Catalogue No. PB513B-1) which contains green fluorescence protein (GFP). *Escherichia coli* Top10 cells were used as maintenance host for the propagation of plasmid. *E. coli* Top10 was grown in Luria-Bertani (LB) broth aerobically at 37°C. Agar plates were made by adding 1.5% (w/v) agar to the liquid media. *E. coli* transformants were selected on LB plates containing 100 μg/ml Ampicillin (Sigma, USA).

### Synthesis and isolation of *LFcinB* gene

The 449 bp full-length bovine lactoferricin was synthesized commercially by Bioneer, Korea, and cloned segment was obtained into the pGEM-B1 vector system. The detection of the *LFcinB* gene into the pGEM-B1 was carried out using detection primers that were 18-mer oligonucleotide (Forward: 5’-CCC TGC TGT CCC TTG GAG-3’) and 20-mer oligonucleotide (Reverse: 5’-TAA AAG GCC CTC CTCA CACA-3’). The supplied vector was linearized using *BamHI* and the targeted gene was isolated (449 bp) by polymerase chain reaction (PCR).

The cloning primers (Forward 5`-ATA GAT CTG ATA TCG CTA GCG AAT TCA AGC-3’ and Reverse 5`-TAG GAT CCG ATA TCG GTA CCG CCG GGC TCG AGC C-3’) containing restriction sites for *EcoRI* and *BamHI* in the forward and reverse primers respectively, were designed for subcloning. PCR amplification was performed using the following programme: 95°C for 5 min, 95°C for 30s, 67°C for 30s and 72°C for 1min, cycle was repeated 35 times, a final extension at 72°C for 10 min. A 50-μL PCR reaction volume was used with a 10 pmole concentration using the Prime Taq DNA polymerase kit (Genet Bio, Korea). PCR products were separated on a 1-2% agarose gel and extracted by Expin Gel DNA gel extraction. The amplified PCR product (1μg) and expression vector PiggyBac (1μg) were digested with 1 μl (20 U/ μl) of the restriction enzymes *EcoRI* and *BamHI*. Two microliters of EZ-One buffer was added in a total reaction volume of 20 μl, incubated at 37 °C for 15 min followed by inactivation at 65 °C for 20 min. The samples that were ready for ligation were kept at -20 °C.

### Cloning of *LFcinB* into mammalian cell-specific expression vector PiggyBac

**Figure 8:**
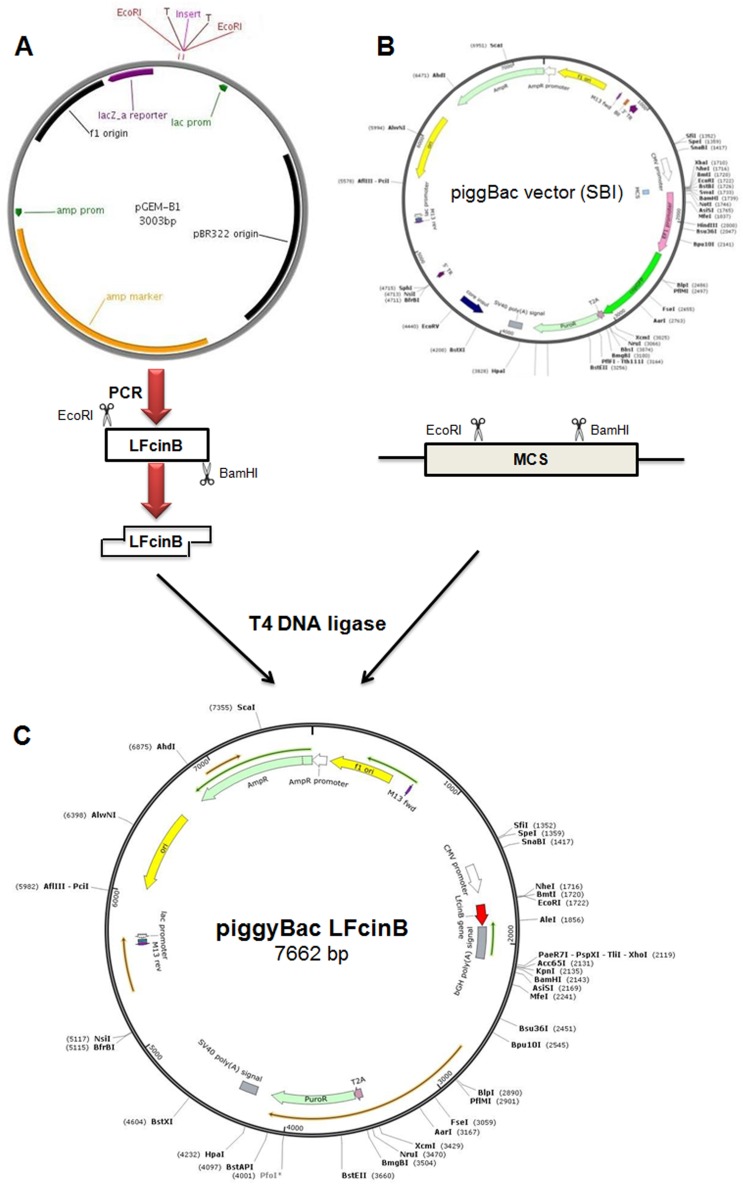
Schematic representation of purification of transgene and construction of recombinant PiggyBac-LFcinB vector **(A)** Isolation of bovine lactoferricin from pGEM-B1 plasmid by double digestion with *EcoRI* and *BamHI* restriction enzymes; **(B)** Targeted PiggyBac cloning vector to construct recombinant vector; **(C)** Constructed recombinant PiggyBac-LFcinB vector (7662 bp).

The strategy for the construction of the mammary-specific expression vector PiggyBac harboring recombinant *LFcinB* gene is shown (Figure 8). CMV and EF1 promoters as present in the Piggybac backbone were enough for the expression of lactoferricin and GFP. The ligation reactions were performed using a T4 DNA ligation kit according to the manufacturer’s instructions (Enzynomics, Korea). PCR products, LFcinB flanking with bovine signal peptide and bovine growth hormone polyA was cloned into mammalian expression vector PiggyBac (7258 bp) with a 1:3 molar ratio of vector and insert for ligation at the *EcoRI* and *BamHI* sites. The reaction mixture was incubated overnight (12 h) at 18°C with 1 μL of T4 DNA ligase enzyme (400 U/μL). The reaction was stopped by incubating at 60°C for 20 min. After completing the ligation process, the ligation mixture was immediately transformed into *E. coli* Top10 competent cells with 100 μg/ml ampicillin as the selection marker. After 12-13 h of incubation at 37 °C, a single colony was selected and grown in 3 ml broth at 37 °C for 8 h. The positive transformants were selected by PCR screening.

The plasmid with the desired gene was designated as PiggyBac-LFcinB (Figure 8) and was subjected to ligation confirmation by single and double digestion. The product was evaluated by agarose gel electrophoresis. The clone (PiggyBac-LFcinB) was further confirmed by DNA sequencing (Bioneer Co., Korea). The end product of recombinant PiggyBac-LFcinB was subjected for endotoxin free using 10% Triton X-114 and subsequently used for transfection in the target cells.

### Cell culture, transfection and selection of stable cell line

Bovine mammary epithelial stem cells (bMESCs) were collected from Korean Holstein cows and maintained in tissue culture flasks in Dulbecco’s Modified Eagle’s Medium/F12 (DMEM/F12) supplemented with 10% fetal bovine serum, 5 μg/ml insulin, 10 ng/ml epidermal growth factor (EGF), 10 ng/ml basic fibroblast growth factor (bFGF), 100 unit/ml penicillin, 100 μg/ml streptomycin, 100 μg/ml gentamicin and 2.5 μg/ml amphotericin B with the seeding rate of 1x10^5^/25 cm^2^ (4000 cells/cm^2^) tissue culture flask [50].

The cells (bMESCs) were subcultured for 2-3 days after attaining the confluency of approximately 90% and fresh medium was added 24 h before transfection. Trypsinized 90% confluent cultured cells were washed twice with phosphate buffer saline (PBS, pH 7). 1-2 x 10^6^ cells were transfected with linearized PiggyBac-LFcinB plasmid (10 μg) using Amaxa Human mammary epithelial cells (HMEC) nucleofector™ kit, and the supplied protocol was followed after some modifications. The ratio of PiggyBac transposon vector clone and PiggyBac transposase vector (PB210PA-1) was optimized as 2.5:1 for the successful transfection. Nucleofection was performed using U-029 program of Amaxa Nucleofector-II system and 5 μg Pmax Green Fluorescent Protein (GFP) vector (Lonza, Amaxa) containing *GFP* gene was used as positive control. bMESCs are very hard to transfect, hence current study optimized dimethyl sulfoxide (DMSO, Sigma, USA) concentration and used as 1.6% in transfection reagent and for 24 h after the pulse. After nucleofection, cells were cultured in the DMEF/F12 growth medium supplemented with 20% FBS. After 48 h of transfection, cells were incubated with 250 μg/ml puromycin for 2-3 weeks. Pure, stable cell lines were stored in DMEM/F12 supplemented with 20% FBS and 10% DMSO for further analysis.

### Detection of *LFcinB* into stable cell lines by immunocytochemistry

Transfected bMESCs were seeded in a 24-well plate, and at 60% confluence were used for immunostaining. Cells were fixed in 4% paraformaldehyde for 30 min at 4°C, then treated with 0.2% Triton X-100 for 30 min at 4°C, followed by treatment with 0.3% H_2_O_2_ for 10 min to stop endogenous peroxidase activity. To prevent non-specific binding, cells were incubated with 10% goat serum for 1 h at 4°C. After they had been washed thrice with PBS, the cells were subjected to immunostaining with Mouse monoclonal Lactoferricin B antibody (MyBiosource.com) primary antibody supplemented at a dilution of 1:5 and incubated for overnight at 4 °C in the dark humidity area. For visualization, fluorescence secondary antibodies for goat anti-mouse conjugated with Phycoerythrin (PE; Santa Cruz, USA) was added at a dilution of 1:50. To visualize nuclei, the cells were counterstained with 0.3 μg/ml 4, 6-diamidino-2-phenylindole (DAPI) (Sigma, USA) for 10 min. Immunostained cells were observed under a fluorescence microscope (Olympus, Japan).

### *LFcinB* secretion ability of transfected bovine mammary epithelial stem cells

The bovine lactoferricin secretion ability of bMESCs was confirmed by the estimation of lactoferricin concentration in the cultured medium of lactoferricin transfected and non-transfected cells as a control. The transfected and non-transfected bMESCs (1 × 10^5^ cells/well) were cultured in growth medium (DMEM/F12) contained in 24-well plate. Media from both the transfected and non-transfected cells was harvested after 3 days of culture and concentration of lactoferrin was determined using the bovine lactoferrin ELISA kit (Cusabio). Media from non-transfected cells was used as a control.

### Bacterial culture and antibacterial activity of *LFcinB*

Bovine mastitis causing bacteria *Staphylococcus aureus* and *Escherichia coli* were isolated directly from clinical cases of bovine mastitis milk using standard protocols of isolation and characterization of isolated bacterial species. After species had been confirmed, the antibacterial activity of LFcinB against both bacteria was evaluated by various antimicrobial assays. All the tests were conducted in triplicates, and results are expressed as mean ± standard deviation (SD).

To test the antimicrobial activity of LFcinB, FBS and antibiotic-free media from the transfected bMESCs were analyzed for antibacterial activity. The antimicrobial activity was determined by agar-well diffusion method [51, 52]. *S. aureus* and *E. coli* were grown in Brain Heart Infusion (BHI) broth and incubated for 24 h at 37 °C. 100 μl of bacterial suspension containing 1 × 10^5^ CFU/ml of each bacterium was spread over the Mueller–Hinton Agar medium, 10 mm wells were prepared and filled with 100 μl media from transfected cells. Penicillin-streptomycin (50 μg/ml) was used as positive control for *S. aureus* and gentamicin (50 μg/ml) for *E. coli*, and media from non-transfected cells were used as negative controls in both the assays. Petri dishes inoculated with the microorganism and containing test samples were incubated at 34 °C for 24 h. The antibacterial activity was determined by measuring the zone of inhibition (in mm) around each well. The zone of inhibition measuring ≤10 mm is considered resistant and higher than that as susceptible [53].

A kinetic study of the antimicrobial activity of recombinant LFcinB against major mastitis causing bacteria was conducted by broth micro-well assay, following the protocol described by Lourenco and Pinto [54] after slight modifications. The wells in a 96 well plate were inoculated with 100 μl containing ∼1 x 10^5^ CFU of *S. aureus* and *E. coli* bacteria and media from transfected and non-transfected bMESCs followed by incubation at 37°C for 24 h in BHI broth. The plates were analyzed for turbidity by measuring the optical density at 595 nm by using a microplate reader (Model-680, Bio-Rad). Penicillin-streptomycin (100 μg/ml) and gentamicin (50 μg/ml) were used as positive controls for *S. aureus* and for *E. coli* respectively, while media from non-transfected were used as negative controls in both assays.

### Statistical analysis

All experiments were performed in triplicates and data were expressed as the mean ± standard deviation (SD). All analyzes were performed using SPSS 16 (SPSS Institute, Cary, NC, USA); individual comparisons were made using Tukey's multiple-range test, which was used to determine the differences between the means. *P*- value of < 0.05 were considered significant.

## Abbreviations

LFcinB: bobine lactoferricin
bMESCs: bovine mammary epithelial stem cells
ELISA: enzyme-linked immunosorbent assay
*S. aureus*: *Staphylococcus aureus*
*E. coli*: *Escherichia coli*
LF: Lactoferrin
AMPs: antimicrobial peptides
BLASTn: balignment search tool-nucleotide
GFP: green fluorescent protein
iPSCs: induced pluripotent stem cells
LPS: lipopolysachharide
LFcin: lactoferricin
NMR: nuclear magnetic resonance
rhLf: recombinant human lactoferrin
BHI: brain heart infusion
FBS: fetal calf serum
PCR: polymerase chain reaction
DMEM/F12: Dulbecco’s Modified Eagle’s Medium/F12
LB: luria-bertani
CMV: cytomegalovirus
EGF: epidermal growth factor
bFGF: basic fibroblast growth factor
bp: basepair
DNA: deoxyribonuclic acid
RNA: ribouclic acid
HMEC: human mammary epithelial cells
PE: phycoerythrin
DAPI: diamidino-2-phenylindole
SD: standard deviation
